# Self-reported insomnia symptoms are associated with urinary incontinence among older Indian adults: evidence from the Longitudinal Ageing Study in India (LASI)

**DOI:** 10.1186/s12889-023-15472-7

**Published:** 2023-03-23

**Authors:** Siqi Leng, Yuming Jin, Michael V. Vitiello, Ye Zhang, Rong Ren, Lin Lu, Jie Shi, Xiangdong Tang

**Affiliations:** 1 Sleep Medicine Center, Department of Urology, Department of Respiratory and Critical Care Medicine, Mental Health Center, West China Hospital, Sichuan University, Dian Xin Nan Jie 28#, Chengdu, 610041 China; 2 Department of Psychiatry and Behavioral Sciences, University of Washington School of Medicine, Seattle, WA USA; 3 National Institute On Drug Dependence and Beijing Key Laboratory of Drug Dependence Research, Peking University, Beijing, 100191 China

**Keywords:** Aging, Insomnia, Urinary incontinence, Stress urinary incontinence, India

## Abstract

**Background:**

Insomnia and urinary incontinence (UI) are both diseases burdening older adults. However, the association between them has not been well elucidated. The purpose of this study is to assess the correlation between insomnia symptoms and UI in a large community‐dwelling sample of older Indian adults.

**Methods:**

Data were from Wave 1 (2017–2018) of the Longitudinal Ageing Study of India (LASI). Male and female participants aged ≥ 60 years who provided complete information on insomnia symptoms, UI, stress UI (SUI), and covariates were included. Insomnia symptoms were identified by a report of: trouble falling asleep, waking up at night, or waking too early, ≥ 5 times/week. UI was defined by self-reported diagnosis. SUI was identified by self-report of involuntary urine leakage when sneezing, coughing, laughing, or lifting weights. Multivariable logistic regression analyses evaluated the associations between insomnia symptoms and UI and SUI. Stratified linear regression evaluated for interactions in prespecified subgroups.

**Results:**

Twenty-six thousand eight hundred twenty-one LASI participants met entry criteria. 2979 (11.11%) reported insomnia symptoms, 976 (3.64%) UI, and 2726 (10.16%) SUI. After full adjustment, insomnia symptoms were associated with both UI and SUI among males (OR 1.53; 95%CI 1.20–1.96 and OR 1.51; 95%CI 1.25–1.83) and females (OR 1.53; 95% CI 1.21–1.92 and OR 1.50; 95% CI 1.31–1.73). A significant interaction effect by age was observed between insomnia symptoms and SUI among both males (*p* = 0.048) and females (*p* = 0.042).

**Conclusions:**

Insomnia symptoms were associated with UI and with SUI in both male and female older Indian adults. Further prospective study is called for to better characterize these associations and to explore underlying mechanisms.

## Introduction

Increasing life expectancy and falling fertility rates have led to older adults becoming a rapidly expanding portion of the Indian population. More than 316 million adults aged 60 years or older are projected in India by 2050, representing 19.1% of the total population [1]. This increasing proportion of older adults is a global phenomenon and with it comes rises in age-related physiological and pathological changes, and age-related diseases [2]. Insomnia, a patient-reported complaint of difficulties in falling asleep, maintaining sleep or early morning awakening, accompanied by adverse daytime consequences [3], is a very common health concern, afflicting 15% of older adults in India [4, 5]. Growing evidence has documented that insomnia is associated with and a risk factor for diseases such as metabolic syndrome, nocturia, sarcopenia, depression, and neurodegenerative disorders including Alzheimer's disease [6–8].

Urinary incontinence (UI) also increases in prevalence with age [9, 10]. UI is common among older adults and results in physical, psychological, and social adverse consequences, contributing to functional limitations and decreased quality of life. Patients living with UI experience restriction of normal activities of daily living, resulting in physical discomfort, emotional burdens of shame and embarrassment, and social isolation [11]. Moreover, older adults with UI are likely to be functionally dependent, leading to heavy caregiver burden and unmet healthcare need [12]. Given that UI patients experience persistent symptoms for more than 10 years and that the condition can worsen over time, the economic burden of this disease is substantial [13, 14]. The prevalence of UI among Indian women is about 12%, while the prevalence of UI among men is less often measured in Indian population [9, 15, 16]. The prevalence of UI in community-dwelling older men in a systematic review is 11%-34%, and the prevalence in older women was 1.3–2.0 times that of older men [17]. UI commonly presents as stress UI (SUI), urgency UI (UUI), overflow UI (OUI), and mixed UI (MUI) [18]. SUI, defined as involuntary leakage of urine on effort or physical exertion, or on sneezing or coughing, is reported to be the most common subtype by most previous UI studies in India [19].

Studies have reported on the association of insomnia and various urologic symptoms, such as nocturia, the commonly observed phenomenon of individuals awakening and realizing that they have to void, which can be a cause of insomnia [20]. However, nocturia is only one of a number of urologic dysfunctions that may be associated with insomnia [21]. Insomnia contributes to neurodegeneration and endocrine dysfunction in ways similar to those seen in ageing, suggesting that insomnia may contribute to the frequency and the severity of age-related chronic disorders, such as UI [22]. However, there is limited evidence demonstrating the link between insomnia symptoms and UI, and even fewer studies have reported the potential relationship between insomnia symptoms and SUI. Considering the sex differences in anatomical structures, risk factors, causes as well as pathophysiological mechanism of UI/SUI, we evaluated the relationships between insomnia symptoms and UI and SUI, separately for males and females, employing the nationally representative data from Wave 1 of the Longitudinal Ageing Study in India (LASI) [10, 23]. We hypothesized that compared to participants without insomnia symptoms those with insomnia symptoms would have higher prevalence rates of UI and SUI.

## Methods

### Data

Data for our study were drawn from wave 1 of LASI, collected from 2017 to 2018. LASI is a representative national study that includes 72,250 individuals aged 45 years and over and their spouses irrespective of age, across all 35 states (except Sikkim) and union territories of India [24]. The LASI database focuses on health and socioeconomic determinants and consequences of the aging process. The survey utilized a multi-stage clustering sampling design. Detailed study methods and microdata for LASI can be accessed at https://lasi-india.org and https://g2aging.org [25]. Ethical approvals and necessary guidelines were approved by The Indian Council of Medical Research (ICMR, Delhi) and the International Institute for Population Sciences (IIPS, Mumbai) with participant informed consent obtained prior to wave 1 survey data collection.

**Fig. 1:**
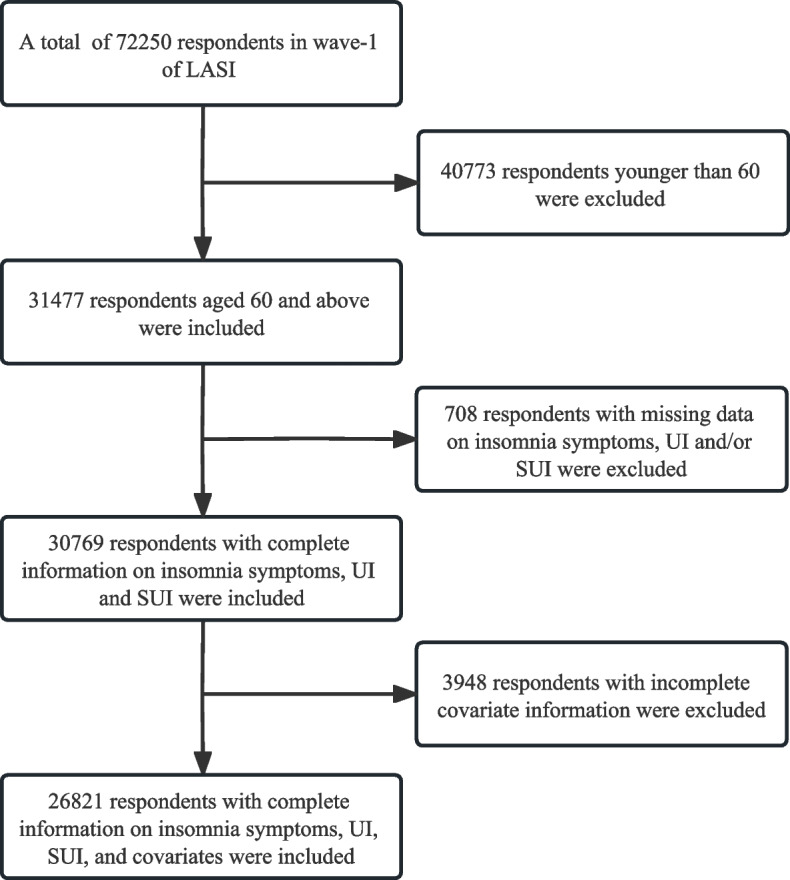
Sample selection for the study. LASI, Longitudinal Ageing Study of India; UI, urinary incontinence; SUI, stress urinary incontinence

Our study was restricted to the data of older adults aged ≥ 60 years, which included 26,821 eligible participants (12,717 males and 14,104 females). We excluded individuals with incomplete information on insomnia symptoms (*n* = 22), on diagnosed UI and/or SUI (*n* = 686), and missing data on covariates (*n* = 3948) (Fig. 1).

### Insomnia symptoms

Detailed data about insomnia symptoms were collected, including three symptoms: 1) trouble falling asleep; 2) waking up at night and having trouble getting back to sleep; 3) waking too early in the morning and not being able to fall asleep. Those three symptoms were selected on the basis of previous studies and were in line with the clinically diagnosed insomnia [26–28], correspondingly, difficulty in initiating sleep, difficulty in maintaining sleep, and early morning awakening were analyzed. The frequency of insomnia symptoms was also recorded, as rarely or never (0–2 times per week), occasionally (3–4 times per week), and frequently (5 or more times per week). We defined a participant as having insomnia symptoms based on their report of at least one of the three insomnia symptoms occurring five or more times per week.

### Diagnosed UI and SUI

UI was defined based on self-report of whether respondents had ever been diagnosed with UI.

SUI was defined based on self-report of respondents ever having passed urine while sneezing, coughing, laughing, or lifting heavy objects.

### Covariates

To ensure that our results are representative and be applied to a wide range of individuals, we adjusted for several potential covariates including sociodemographic and biological factors.

Sociodemographic variable*s* included; age in years, sex, level of education (no schooling/less than 5 years complete/5–9 years complete/10 or more years complete), working status (currently unemployed/currently employed), marital status (married or partnered/widowed/others), living arrangement (co-residential living/separate living, “co-residential living” for “living with spouse, children or other household member”, and “separate living” for “living alone”), place of residence (urban/rural), economic status (low/middle/high, trichotomized by annual per capita household consumption. Annual per capita household consumption was used as a proxy for economic status based on prior studies [29, 30]. Household consumption included self-reported expenditure on food, household utilities, fees, durable goods, education, transit, remittances, and discretionary spending and outpatient and inpatient health care in the previous year. Annual per capita household consumption was calculated by taking household consumption divided by the total number of household members), religion (Hindu/Muslim/Christian/others) and caste (scheduled caste/scheduled tribal/other backward class/none of the above).

Insomnia symptoms were associated with underweight, chronic diseases and physically inactivity based on prior study using LASI data [26].Thus, we included biological factors, i.e., body mass index (BMI), waist-to-hip ratio, frequency of vigorous physical activity, number of chronic diseases, medication/treatment status, self-rated health (SRH), drinking status, smoking status, depression, and pain. Medication/Treatment status was categorized into “no” for “never having taken medication or used other treatments to help sleep” and “yes” for “having taken medication or used other treatments to help sleep”. BMI was categorized as < 18.5, 18.5–25, 25–29.9, ≥ 30 kg/m^2^. Waist-to-hip ratio was dichotomized into low risk (< 0.90 for male, while < 0.85 for female) and high risk (≥ 0.90 for male, while ≥ 0.85 for female). Vigorous physical activity was about respondents’ involvement in running or jogging, swimming, going to a health center/gym, cycling, digging with a spade or shovel, heavy lifting, chopping, farm work, fast bicycling, and cycling with loads and was classified by frequency as “every day”, “more than once a week”, “once a week”, “1–3 times a month”, “hardly ever or never”. Number of chronic diseases included self-reported hypertension, diabetes, tumor, lung disease, chronic heart disease, stroke, arthritis, mental disease, Alzheimer's disease, hypercholesterolemia, asthma, congestive heart failure, heart attack, abnormal heart rate, osteoporosis, abnormal thyroid function, digestive disease, skin disease, kidney stone, presbyopia, cataract, glaucoma, myopia, hyperopia, tooth decay, and periodontal disease. The variable was recorded as “0” if the respondent did not have any chronic disease, “1”, if the respondent had only one chronic disease and “2 + ” if the respondent had more than two chronic diseases. SRH was sorted by a five‐point Likert scale as “excellent,” “very good,” “good,” “fair” and “poor”, which was a proxy indicator for health status. Drinking status, defined as consumption of any form of alcohol in one’s lifetime, was categorized into “no” for “never having had an alcoholic drink” and “yes” for “currently or ever having consumed any alcoholic beverages.” Smoking status, defined as consumption any form of tobacco in one’s lifetime, was categorized into “no” for “never having smoked” and “yes” for “current or ever having smoked.” Depression was dichotomized into “no” for “not diagnosed with depression” and “yes” for “diagnosed.” The presence of depression was evaluated using internationally validated 10-item Center for Epidemiologic Studies Depression Scale (CESD-10), in which score ≥ 4 out of overall 10 score was defined as depression) [31]. Pain was divided into “no” for “not troubled with pain” and “yes” for “often troubled with pain.”

### Statistical analysis

We expressed continuous variables as mean and standard deviation and categorical variables as proportions. Kruskal Wallis H test (continuous variables) and chi-square tests (categorical variables) were used to calculate statistical differences in baseline characteristics among groups dichotomized by whether reporting insomnia symptoms. Besides, we conducted multivariate logistic regression dividing the individuals by sex to evaluate the associations between insomnia symptoms and diagnosed UI or SUI. The incremental models were constructed adjusting for covariates: no covariates in the unadjusted model; sociodemographic covariates in model 1 i.e., age, level of education, work status, marital status, living arrangement, place of residence, economic status, religion, and caste. And for the fully adjusted model 2, we adjusted for the sociodemographic mentioned above and for the biological covariates: medication/treatment status, BMI, vigorous physical activity, waist-to-hip ratio, number of chronic diseases, SRH, drinking status, smoking status, depression, and pain.

We performed interaction analyses to evaluate the heterogeneity of association between insomnia symptoms and UI or SUI stratified by covariates (including age, BMI, drinking status, smoking status and medication/treatment status). Given that average life expectancy in India is 69.4 years in 2014–18, age was categorized as < 70 and ≥ 70 years [32]. BMI levels were categorized into underweight (< 18.5 kg/m^2^), normal (18.5 to 24.9 kg/m^2^) and overweight/obese (≥ 25 kg/m^2^) subgroups, since the sample size of obese subjects (BMI ≥ 30 kg/m^2^) was limited. Drinking and smoking status were dichotomized into “yes” and “no” as mentioned above. As a control for potential sex-based differences, our study investigated the interaction separately for males and females. The subgroup analyses were performed using stratified linear regression models, while the p for interaction was calculated using the log-likelihood ratio test to compare the differences between models with and without the interaction of covariates.

All statistical analyses were conducted using the statistical software packages R (http://www.R-project.org, The R Foundation) and Empower (http://www.empowerstats.com). Two-tailed *P*-values were performed with a significance level of < 0.05.

## Results

### Baseline characteristics

The characteristics and related covariates of participants are summarized in Table 1. The prevalence of insomnia symptoms was 11.11%, Patients with insomnia symptoms were more likely to be older, female, less educated, unemployed, unmarried, living separately, living in rural areas, Hindu, lower proportion of high-risk waist-to-hip ratio, physically inactivity, BMI ≥ 30 or < 18.5 kg/m^2^, with two or more chronic diseases, and with poor SRH.

**Table 1:** Baseline characteristics of participants

Characteristics	Total	Insomnia symptoms		
		**No (*n* = 23,842)**	**Yes (*n* = 2979)**	***p*-value**
**Age, year (mean ± SD)**	68.61 ± 7.27	68.50 ± 7.20	69.49 ± 7.72	< 0.001
**Sex, n (%)**				< 0.001
Male	12,717 (47.41%)	11,470 (48.11%)	1247 (41.86%)	
Female	14,104 (52.59%)	12,372 (51.89%)	1732 (58.14%)	
**Level of education, n (%)**				< 0.001
No schooling	14,384 (53.63%)	12,662 (53.11%)	1722 (57.80%)	
Less than 5 years complete	8463 (31.55%)	7540 (31.62%)	923 (30.98%)	
5–9 years complete	2796 (10.42%)	2551 (10.70%)	245 (8.22%)	
10 or more years complete	1178 (4.39%)	1089 (4.57%)	89 (2.99%)	
**Working status, n (%)**				< 0.001
Currently unemployed	17,538 (65.39%)	15,460 (64.84%)	2078 (69.75%)	
Currently employed	9283 (34.61%)	8382 (35.16%)	901 (30.25%)	
**UI, n (%)**				< 0.001
No	25,845 (96.36%)	23,083 (96.82%)	2762 (92.72%)	
Yes	976 (3.64%)	759 (3.18%)	217 (7.28%)	
**SUI, n (%)**				< 0.001
No	24,095 (89.84%)	21,628 (90.71%)	2467 (82.81%)	
Yes	2726 (10.16%)	2214 (9.29%)	512 (17.19%)	
**Medications/Treatments to help sleep, n (%)**				< 0.001
No	26,014 (96.99%)	23,342 (97.90%)	2672 (89.69%)	
Yes	807 (3.01%)	500 (2.10%)	307 (10.31%)	
**BMI, kg/m**^**2**^**, n (%)**				< 0.001
< 18.5	6174 (23.02%)	5367 (22.51%)	807 (27.09%)	
18.5–25	14,094 (52.55%)	12,647 (53.05%)	1447 (48.57%)	
25–30	4996 (18.63%)	4458 (18.70%)	538 (18.06%)	
≥ 30	1557 (5.81%)	1370 (5.75%)	187 (6.28%)	
**Waist-to-hip ratio, n (%)**				< 0.001
Low risk	2851 (10.63%)	2444 (10.25%)	407 (13.66%)	
High risk	23,970 (89.37%)	21,398 (89.75%)	2572 (86.34%)	
**Vigorous physical activity, n (%)**				< 0.001
Everyday	4874 (18.17%)	4432 (18.59%)	442 (14.84%)	
More than once a week	1502 (5.60%)	1375 (5.77%)	127 (4.26%)	
Once a week	855 (3.19%)	766 (3.21%)	89 (2.99%)	
One to three times a month	1200 (4.47%)	1078 (4.52%)	122 (4.10%)	
Hardly ever or never	18,390 (68.57%)	16,191 (67.91%)	2199 (73.82%)	
**Number of chronic diseases, n (%)**				< 0.001
0	4410 (16.44%)	4127 (17.31%)	283 (9.50%)	
1	5771 (21.52%)	5293 (22.20%)	478 (16.05%)	
2 +	16,640 (62.04%)	14,422 (60.49%)	2218 (74.45%)	
**SRH, n (%)**				< 0.001
Excellent	750 (2.80%)	708 (2.97%)	42 (1.41%)	
Very good	3889 (14.50%)	3619 (15.18%)	270 (9.06%)	
Good	9767 (36.42%)	8989 (37.70%)	778 (26.12%)	
Fair	8827 (32.91%)	7751 (32.51%)	1076 (36.12%)	
Poor	3588 (13.38%)	2775 (11.64%)	813 (27.29%)	
**Drinking status, n (%)**				0.91
Never	22,222 (82.85%)	19,756 (82.86%)	2466 (82.78%)	
Current/ever	4599 (17.15%)	4086 (17.14%)	513 (17.22%)	
**Smoking status, n (%)**				0.058
Never	21,394 (79.77%)	19,057 (79.93%)	2337 (78.45%)	
Current/ever	5427 (20.23%)	4785 (20.07%)	642 (21.55%)	
**Depression, n (%)**				< 0.001
No	19,486 (72.65%)	17,846 (74.85%)	1640 (55.05%)	
Yes	7335 (27.35%)	5996 (25.15%)	1339 (44.95%)	
**Pain, n (%)**				< 0.001
No	15,920 (59.36%)	14,618 (61.31%)	1302 (43.71%)	
Yes	10,901 (40.64%)	9224 (38.69%)	1677 (56.29%)	
**Marital status, n (%)**				< 0.001
Married or partnered	17,212 (64.17%)	15,479 (64.92%)	1733 (58.17%)	
Widowed	9068 (33.81%)	7880 (33.05%)	1188 (39.88%)	
Others	541 (2.02%)	483 (2.03%)	58 (1.95%)	
**Living arrangement, n (%)**				0.001
Co-residential living	25,429 (94.81%)	22,641 (94.96%)	2788 (93.59%)	
Separate living	1392 (5.19%)	1201 (5.04%)	191 (6.41%)	
**Place of residence, n (%)**				< 0.001
Urban	8911 (33.22%)	8010 (33.60%)	901 (30.25%)	
Rural	17,910 (66.78%)	15,832 (66.40%)	2078 (69.75%)	
**Economic status, n (%)**				0.357
Low	9570 (35.68%)	8511 (35.70%)	1059 (35.55%)	
Middle	9141 (34.08%)	8153 (34.20%)	988 (33.17%)	
High	8110 (30.24%)	7178 (30.11%)	932 (31.29%)	
**Religion, n (%)**				< 0.001
Hindu	19,700 (73.45%)	17,366 (72.84%)	2334 (78.35%)	
Muslim	3158 (11.77%)	2808 (11.78%)	350 (11.75%)	
Christian	2632 (9.81%)	2475 (10.38%)	157 (5.27%)	
Others	1331 (4.96%)	1193 (5.00%)	138 (4.63%)	
**Caste, n (%)**				< 0.001
Scheduled caste	4410 (16.44%)	3846 (16.13%)	564 (18.93%)	
Scheduled trible	4465 (16.65%)	4146 (17.39%)	319 (10.71%)	
Other backward class	10,287 (38.35%)	9028 (37.87%)	1259 (42.26%)	
Other castes	7659 (28.56%)	6822 (28.61%)	837 (28.10%)	

*SD* standard deviation, *UI* urinary incontinence, *SUI* stress urinary incontinence, *BMI* body mass index, *SRH* self-rated health

Mean ± SD for continuous variables: P value was calculated by Kruskal Wallis H test

Number (%) for categorical variables: P value was calculated by chi-square test

3.64% of respondents reported UI and 10.16% reported SUI. UI was reported by 7.28% of participants with insomnia symptoms compared to 3.18% by participants without insomnia symptoms. SUI was reported by 17.19% of participants with insomnia symptoms and by 9.29% of participants without insomnia symptoms.

### Insomnia symptoms and associated UI and SUI

Results from the multivariable linear regression analysis of insomnia symptoms and UI, and insomnia symptoms and SUI are shown in Table 2. Having separated the respondents by sex, we found that, after adjusting for only sociodemographic covariates (model 1), insomnia symptoms were associated with UI for both male respondents (OR 2.24; 95% CI 1.78–2.83) and female respondents (OR 2.23; 95% CI 1.80–2.77). Further adjustment for health characteristics (model 2) moderately attenuated the association for both sexes (OR 1.53; 95% CI 1.20–1.96 for males, and OR 1.53; 95% CI 1.21–1.92 for females). Similarly, insomnia symptoms were significantly related to SUI symptoms in model 1 for both males (OR 2.13; 95% CI 1.78–2.54) and females (OR 1.90; 95% CI 1.66–2.17). Full adjustment (model 2) attenuated the association for both sexes (OR 1.51; 95% CI 1.25–1.83 for males, and OR 1.50; 95% CI 1.31–1.73 for females).

**Table 2:** Relationship between insomnia symptoms and associated UI and SUI

Insomnia symptoms	OR (95% CI)	
	Male (*n* = 12,717)	Female (*n* = 14,104)
UI		
Unadjusted model	2.50 (1.99, 3.15)	2.33 (1.88, 2.89)
Model 1	2.24 (1.78, 2.83)	2.23 (1.80, 2.77)
Model 2	1.53 (1.20, 1.96)	1.53 (1.21, 1.92)
SUI		
Unadjusted model	2.22 (1.86, 2.64)	1.53 (1.21, 1.92)
Model 1	2.13 (1.78, 2.54)	1.90 (1.66, 2.17)
Model 2	1.51 (1.25, 1.83)	1.50 (1.31, 1.73)

*OR* odds ratio, *95% CI* 95% Confidence interval, *UI* urinary incontinence, *SUI* stress urinary incontinence

Unadjusted model: no covariates were adjusted

Model 1 adjusted for: age, level of education, work status, marital status, living arrangement, place of residence, economic status, religion, caste

Model 2 adjusted for: age, level of education, work status, marital status, religion, place of residence, living arrangement, economic status, caste, medication/treatment status, body mass index (BMI), vigorous physical activity, waist-to-hip ratio, number of chronic diseases, self-rated health (SRH), drinking status, smoking status, depression, pain

### Subgroup analysis

**Fig. 2:**
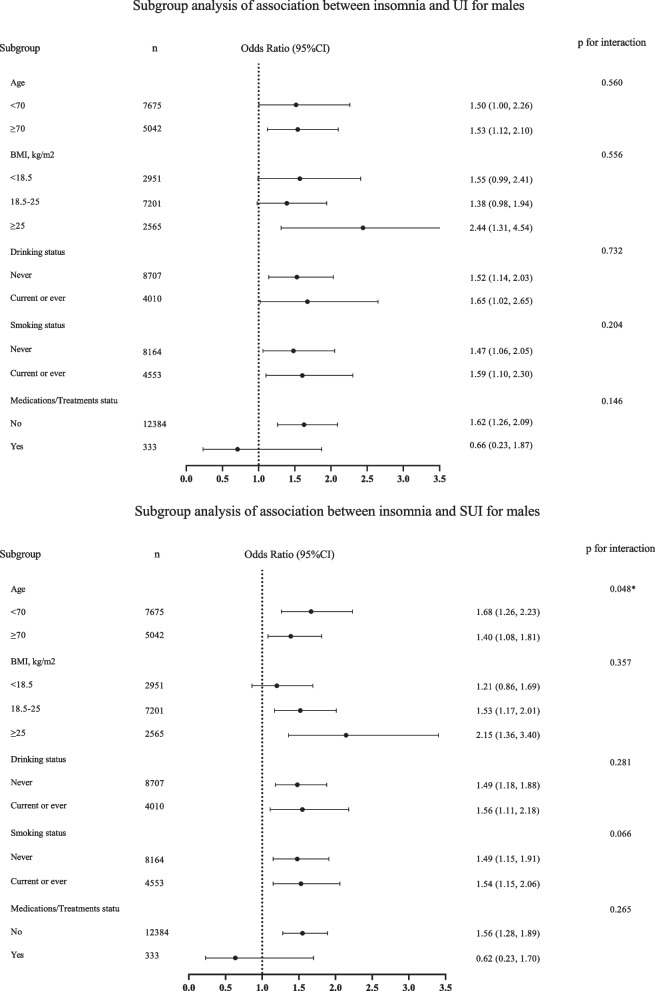

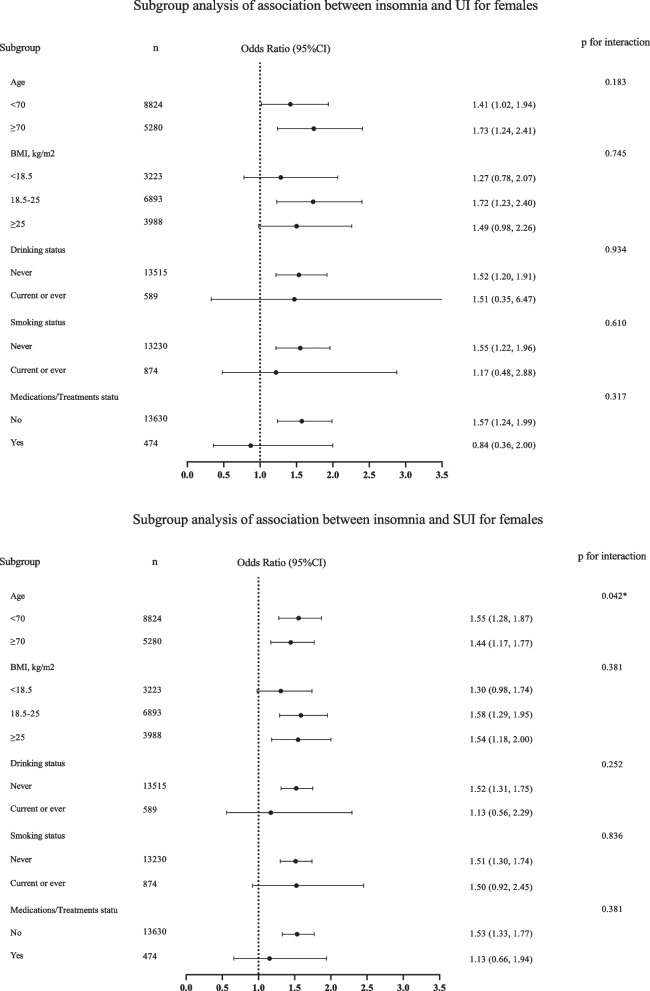
Subgroup analysis of relationship between insomnia and associated UI and SUI. We recoded the BMI and recategorized it into three groups: < 18.5, 18.5–25, ≥ 25 kg/m^2^, due to the limited sample size of BMI ≥ 30 kg/m^2^. OR, odds ratio; 95% CI, 95% Confidence interval; UI, urinary incontinence; SUI, stress urinary incontinence; BMI, body mass index. Model 2 adjusted for: age, level of education, work status, marital status, religion, place of residence, living arrangement, economic status, caste, medication/treatment status, BMI, vigorous physical activity, waist-to-hip ratio, number of chronic diseases, self-rated health (SRH), drinking status, smoking status, depression, pain except the subgroup variable

We conducted interaction tests to further assess the relationship between insomnia symptoms and UI and SUI the results of which are presented in Fig. 2. And we recategorized BMI into three groups: < 18.5, 18.5–25, ≥ 25 kg/m^2^ in subgroup analysis, due to the limited sample size of BMI ≥ 30 kg/m^2^. No significant interactions were found in stratified analyses by age, BMI, drinking status, smoking status and medication/treatment status for the associations between insomnia symptoms and UI for males (*p* = 0.560, 0.556, 0.732, 0.204, 0.146, respectively) or for females (*p* = 0.183, 0.745, 0.934, 0.610, 0.317, respectively). Comparable lack of significant insomnia/SUI interactions were found in stratified analyses for BMI, drinking, smoking and medications/treatments taking for both males (*p* = 0.357, 0.281, 0.066, 0.265, respectively) and females (*p* = 0.381, 0.252, 0.836, 0.381, respectively). However, in the subgroup analysis stratified by age, there was a significant interaction of insomnia symptoms and SUI for both males (*p* = 0.048) and females (*p* = 0.042), with positive associations observed among participants aged both < 70 years (OR 1.68; 95% CI 1.26–2.23 for males, while OR 1.55; 95% CI 1.28–1.87 for females) and ≥ 70 years (OR 1.40; 95% CI 1.08–1.81 for males, and OR 1.44; 95% CI 1.17–1.77 for females).

## Discussion

Insomnia symptoms were present in 11.11% of the study sample and was significantly associated with both diagnosed UI and with SUI in both males and females. These associations remained statistically significant after adjustment for multiple potential covariates. Notably, we did not detect a significant sex difference on the relationship between insomnia symptoms and UI and SUI. moreover, only a single a significant interaction was observed, that of age in the association of insomnia symptoms and SUI in both males and females, with the insomnia/SUI association positive among participants aged < 70 years and ≥ 70 years, which did not differ by sex.

The prevalence of diagnosed UI was 3.64%, and 10.16% respondents reported symptoms of SUI. Diagnosed UI prevalence was lower than that of SUI symptoms. Compared with the relatively strict diagnosis of UI, SUI status was based on reporting ever having experienced any SUI event, a very lax definition which likely resulted in the higher reported prevalence. Moreover, low treatment seeking for UI may also contribute to this discrepancy, which is in line with prior studies, suggesting that UI remains underrecognized and underestimated, with fewer than 40% of affected females seeking treatments to diagnose this disease [33]. Nevertheless, the overall prevalence of UI was relatively lower that reported in several small-sample studies focusing on the general Indian population [15, 16]. It can be interpreted by the fact that there is wide variability in UI prevalence estimates (5% to 70%) from various countries, depending on the definitions of UI used, the study population, and the assessment tools and availability of health care, and with higher prevalence rates reported in western countries [9, 34, 35].

Inconsistent relationships between sleep quality and UI have been reported in earlier studies. Siddiqui et al. carried out cross-sectional analysis on a sample of 510 treatment seeking females with UI, finding out that there was no difference in sleep quality based on the presence and severity of urinary incontinence after adjustment [36]. Dasdemir Ilkhan et al. reported that sleep status was not associated with UI and incontinence-related life quality among 1150 older adults residing in nursing homes in Istanbul (*P* > 0.05) [37]. In contrast, Araujo et al. conducted a prospective cohort study of 4145 individuals, reported a bi-directional link between sleep-related problems and UI, with BMI possibly mediating the relationship [21]. Ge et al. in a study of fifty-one overactive bladder (OAB) patients, reported a positive correlation between sleep quality and UI status [38]. Yilmaz Bulut et al. found a similar association between UI symptoms and sleep quality among 140 older females lived in Turkey [39].

One possible explanation of our finding is that mental factors could mediate the association between insomnia symptoms and UI. Several longitudinal studies and meta-analyses have identified insomnia as a risk factor for depression, anxiety, and other mental disorders among adults [40, 41]. It is conceivable that insomnia’s adverse impact on mental health may affect symptoms of UI. This is supported by studies reporting that depression is related to UI symptom severity, functional impairment, and incontinence-related life quality [42]. In addition, the negative emotional impacts caused by UI can in turn contribute to sleep disturbance, given that the association between insomnia and psychological distress is bidirectional [43, 44]. Finally, insomnia is also accompanied by daytime function difficulties and cognitive decline, which may further contribute to the burden of UI, making it more difficult for UI patients to lose weight, reduce caffeine and nicotine intake, and conduct pelvic floor muscle training [45, 46].

Another possible link between insomnia symptoms and UI is metabolic disturbance. Acute and chronic sleep deprivation is associated with metabolic disorders, which influence hormonal secretion patterns, autonomic nervous system balance and vasopressin secretion [22, 47]. These pathways each impact the regulation of smooth muscle tone, which is fundamental to relaxation/contraction of the detrusor and bladder musculature, and thus may be linked with urination function [21]. Additionally, obesity and related metabolic disorders are also related to systemic inflammation, pro-inflammatory cytokine release and oxidative stress, thereby altering collagen metabolism, accompanied with increased intra-abdominal pressure, leading to the progression of SUI [48, 49].

Accumulating evidence also suggests a possible neuro-molecular mechanism underlying the association between insomnia symptoms and UI and SUI. Disturbed sleep, which has long been considered a symptom of neurodegenerative conditions, may in fact be a risk factor for and trigger the onset of these diseases in the early stages via processes such as endoplasmic reticulum stress and neuronal damage [50–52]. Studies on the molecular mechanism of SUI have shown that SUI is related to the differential expression of neuronal cell-specific proteins and neurodegeneration-related proteins, which indicates the potential involvement of a neurodegeneration process in SUI [53]. We speculate that potential neuropathological effects of insomnia symptoms may be related to the development of SUI.

Our study has clear limitations. Given that it is cross-sectional, only associational and not causal relationships can be inferred. Future waves of data collection in LASI will allow for identification of potential causal relationships. Monthly recall of insomnia symptoms can only provide short-term information and may not reflect participants’ usual sleep status, while the UI and SUI status can provide long-term information based on ever having been diagnosed UI or experienced any SUI event. Thus, the mismatch of time timeframe limits our ability to identity the direction of causality for the relationships we studied. Self-report of health conditions, including insomnia symptoms, UI, and SUI, are subject to recall bias, idiosyncratic interpretation of the question and other reporting errors. An additional limitation the lack of severity and duration measures for insomnia symptoms, UI and SUI, such that the contribution of severity and duration to associations of insomnia symptoms and UI and SUI could not be assessed. An analytic limitation is that data analyses were limited to variables collected in the parent study, which limited our ability to define better insomnia symptoms and SUI, as well as prohibited the examination of other forms of UI. Finally, the LASI database, although extensive, limited the number of potential co-variates available for analysis.

Nevertheless, our study also has several strengths. Data in our study were collected from a large nationally representative sample, using standardized processes and protocols to ensure high quality data. To the best of our knowledge, this is the first attempt to explore the relationships between insomnia symptoms and UI and SUI among older Indian males and females. To ensure that our results were nationally representative our data analyses were adjusted for multiple potential covariates. After full adjustment, the observed associations between insomnia symptoms and UI and SUI remained unchanged, although their magnitudes were diminished, which suggests that the study’s findings are robust with results that are stable and reliable. Additionally, our results indicate the need for future longitudinal study of the association between insomnia symptoms and UI and SUI to determine its directionality and explore potential underlying mechanisms.

## Conclusions

Insomnia symptoms were associated with greater prevalence both of diagnosed UI and of SUI among older Indian males and females, independent of covariates. It can be recommended that older adults with insomnia symptoms need early screening and appropriate treatment of UI and SUI, which improve their quality of life and gain in public health benefits in low- and middle-income countries, as an unprecedented pace of population aging continues. And further research is called for to better characterize this association, determine underlying mechanisms and to explore the potential therapeutic implications.

## Data Availability

The datasets generated and/or analysed during the current study are available in the [Gateway to Global Aging Data] repository, [https://lasi-india.org].

## Abbreviations

LASI: Longitudinal Ageing Study in India
UI: Urinary incontinence
SUI: Stress urinary incontinence
BMI: Body mass index
SRH: Self-rated health
OR: Odds ratio
95% CI: 95% Confidence interval
