# Correction: Self-reported insomnia symptoms are associated with urinary incontinence among older Indian adults: evidence from the Longitudinal Ageing Study in India (LASI)

**DOI:** 10.1186/s12889-023-15594-y

**Published:** 2023-06-27

**Authors:** Siqi Leng, Yuming Jin, Michael V. Vitiello, Ye Zhang, Rong Ren, Lin Lu, Jie Shi, Xiangdong Tang

**Affiliations:** 1 Sleep Medicine CenterDepartment of Urology, Department of Respiratory and Critical Care Medicine, Mental Health CenterWest China Hospital, Sichuan University, Dian Xin Nan Jie 28#, Chengdu, 610041 China; 2 Department of Psychiatry and Behavioral Sciences, University of Washington School of Medicine, Seattle, WA USA; 3 National Institute On Drug Dependence and Beijing Key Laboratory of Drug Dependence Research, Peking University, Beijing, 100191 China

**Correction** **:** **BMC Public Health 23, 552 (2023)**

**https://doi.org/10.1186/s12889-023-15472-7**

In the original publication of this article [1] there was an error in Fig. 2, the top and bottom part of the figure were identical. The updated figure (Fig. 1) is available in this correction article. The original article has been updated.

**Fig. 2:**
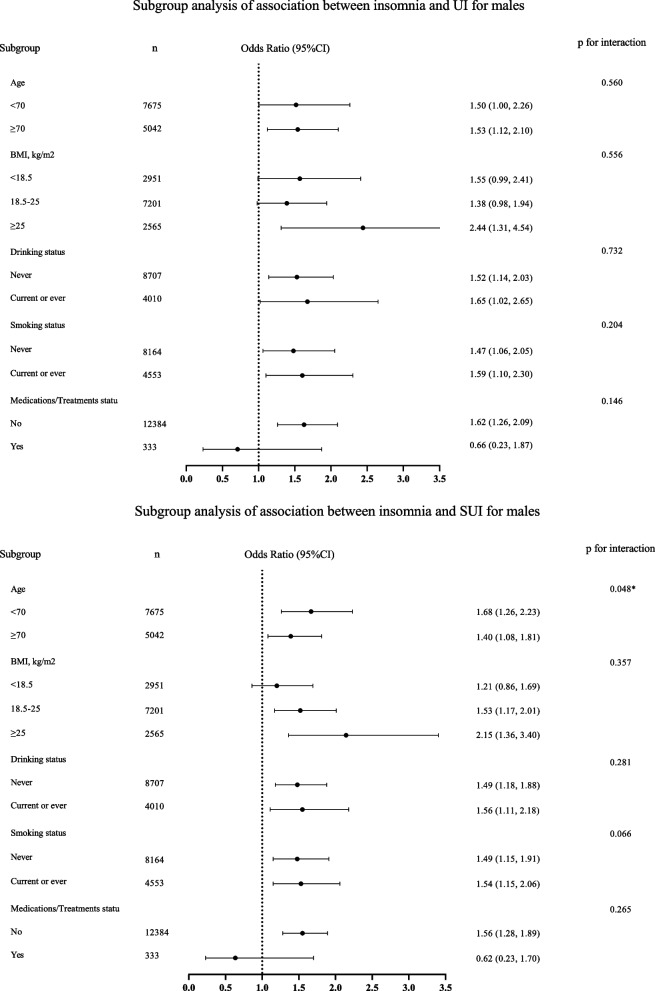

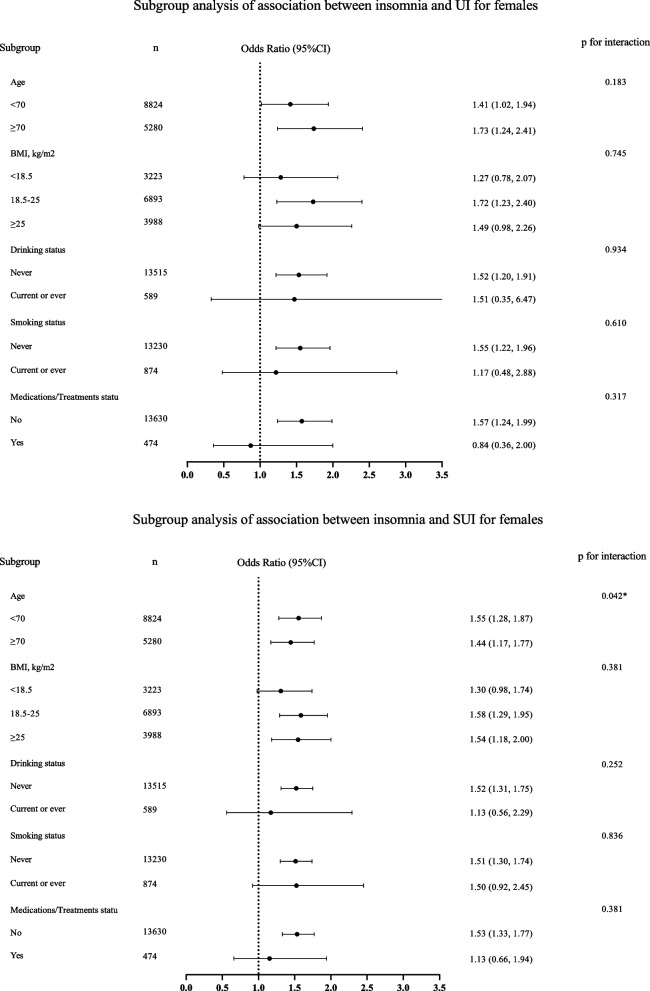
Subgroup analysis of relationship between insomnia and associated UI and SUI. We recoded the BMI and recategorized it into three groups: < 18.5, 18.5–25, ≥ 25 kg/m^2^, due to the limited sample size of BMI ≥ 30 kg/m^2^. OR, odds ratio; 95% CI, 95% Confidence interval; UI, urinary incontinence; SUI, stress urinary incontinence; BMI, body mass index. Model 2 adjusted for: age, level of education, work status, marital status, religion, place of residence, living arrangement, economic status, caste, medication/treatment status, BMI, vigorous physical activity, waist-to-hipratio, number of chronic diseases, self-rated health (SRH), drinking status, smoking status, depression, pain except the subgroup variable

Fig. 1 correct version of Fig. 2
